# Characteristics of viral pneumonia in non-HIV immunocompromised and immunocompetent patients: a retrospective cohort study

**DOI:** 10.1186/s12879-021-06437-5

**Published:** 2021-08-06

**Authors:** Lijuan Li, Steven H. Hsu, Chunlei Wang, Binbin Li, Lingxiao Sun, Jinying Shi, Yali Ren, Jinxiang Wang, Xiaoqi Zhang, Jiangbo Liu

**Affiliations:** 1 Department of Pulmonary and Critical Care Medicine, National Center for Clinical Research on Respiratory Diseases, China-Japan Friendship Hospital, 2 Yinghuayuan E St, Chaoyang District, Beijing, 100029 China; 2 Department of Medicine, Houston Methodist Hospital, Houston, TX 77030 USA; 3 Clinical Center for Pulmonary Infections, Capital Medical University, Beijing, 100029 China; 4 Department of Pulmonary and Critical Care Medicine, First Hospital of Shijiazhuang, Shijiazhuang, 050011 China; 5 Department of Pulmonary and Critical Care Medicine, Second Hospital of Hebei Medical University, Shijiazhuang, 050000 China; 6 Department of Respiratory and Critical Care Medicine, Beijing Luhe Hospital of Capital Medical University, Beijing, 101100 China; 7 Department of Pulmonary and Critical Care Medicine, Second People’s Hospital of Weifang, Weifang, 261041 China; 8 Department of Pulmonary and Critical Care Medicine, Tianjin First Central Hospital, Tianjin, 300192 China

**Keywords:** Viral pneumonia, Immunocompromised, Immunocompetent, Prognosis

## Abstract

**Background:**

Concerning viral pneumonia, few large-scale comparative studies have been published describing non-HIV immunocompromised and immunocompetent patients, but the epidemiological characteristics of different viruses or underlying diseases in immunocompromised hosts are lacking.

**Methods:**

We retrospectively recruited patients hospitalised with viral pneumonia from six academic hospitals in China between August 2016 and December 2019. We measured the prevalence of comorbidities, coinfections, nosocomial infections, and in-hospital mortalities.

**Results:**

Of the 806 patients, 370 were immunocompromised and 436 were immunocompetent. The disease severity and in-hospital mortality of immunocompromised patients were higher than those of immunocompetent patients. During the influenza season, an increased number of cases of influenza virus (IFV) infection were found in the immunocompromised group, followed by cases of cytomegalovirus (CMV) and respiratory syncytial virus (RSV) infection. During the non-influenza season, CMV was the main virus detected in the immunocompromised group, while RSV, adenovirus (AdV), parainfluenza virus (PIV), and rhinovirus (HRV) were the main viruses detected in the immunocompetent group. Pneumonia caused by *Pneumocystis jirovecii* (22.4%), *Aspergillus* spp. (14.1%), and bacteria (13.8%) were the most frequently observed coinfections in immunocompromised patients but not in immunocompetent patients (*Aspergillus* spp. [10.8%], bacteria [7.1%], and *Mycoplasma* spp. [5.3%]). CMV infection and infection with two-or-more viruses were associated with a higher in-hospital mortality rate than non-IFV infection. However, patients with IFV and non-IFV infection in immunocompromised patients had similar disease severity and prognosis.

**Conclusions:**

Immunocompromised patients have a high frequency of coinfections, and a higher mortality rate was observed among those infected with CMV and two-or-more viruses. In addition, patients with IFV and non-IFV infection in immunocompromised patients had similar same disease severity and prognosis. The type of viral infection varied with seasons.

**Supplementary Information:**

The online version contains supplementary material available at 10.1186/s12879-021-06437-5.

## Background

Among transplant recipients and patients with haematological malignancy, viral pneumonia often leads to severe respiratory disease and death [1]. Viral lower respiratory tract infections in immunocompromised patients have generally been ascribed to herpes virus (HSV) and cytomegalovirus (CMV) [2]. In recent years, influenza virus (IFV), parainfluenza virus (PIV), respiratory syncytial virus (RSV), and rhinovirus (HRV) have also been recognised as causes of serious infections, especially in patients undergoing treatment for haematologic malignancies and haematopoietic stem cell transplantation. These patients have a higher tendency to develop severe pneumonia, and a mortality rate as high as 25–70% has been reported [3–7]. These patients might experience prolonged viral shedding that potentially result in a longer duration of infection, a higher nosocomial transmission rate, and a higher mortality rate than those of immunocompetent hosts [8, 9]. Concerning non-HIV immunocompromised patients with viral pneumonia, few large-scale epidemiological studies and comparative studies have investigated different viruses or underlying diseases; however, investigations on the epidemiological and etiologic characteristics are lacking.

The objective of this study was to examine the epidemiological and etiologic characteristics and to identify the most common types of viruses that cause viral pneumonia in non-HIV immunocompromised and immunocompetent patients.

## Methods

### Study design and participants

We retrospectively recruited patients with community-acquired pneumonia (CAP) who were hospitalised between August 2016 and December 2019 at one of the six secondary and tertiary academic hospitals in China. The diagnosis of CAP was based on the American Thoracic Society and Infectious Disease Society of America (ATS/IDSA) guidelines [10]. Immunocompromised patients were selected if they met any of the following inclusion criteria: (1) solid-organ, stem cell, or bone marrow transplant recipients; (2) undergoing chemotherapy for any haematological disease (including acute lymphocytic leukaemia, acute myeloid leukaemia, chronic lymphocytic leukaemia, myeloma, or lymphoma) or the presence of a solid tumour within 6 months of admission or neutropenia (neutrophil count < 500 cells/mm^3^); (3) chest radiation therapy within 3 months of admission; (4) an autoimmune disease (including but not limited to systemic lupus erythematosus, rheumatoid arthritis, polymyalgia rheumatica, and interstitial lung disease) and receiving immunosuppressive therapy (including chronic glucocorticoid treatment: oral prednisone > 10 mg/d or the equivalent for ≥3 weeks) or methotrexate > 12.5 mg/week, cyclosporine, azathioprine, or biological modifiers such as etanercept or infliximab within 3 months of admission; and (5) history of splenectomy or cirrhosis [1, 11, 12]. Patients were excluded if they (1) were aged < 14 years, (2) experienced pneumonia onset ≥48 h after admission, or (3) tested positive for human immunodeficiency virus.

### Study quality control

Key investigators, including clinicians, statisticians, microbiologists, and radiologists, worked together to draft the protocol and created a single formatted case report form (CRF) that was used by all centres. Before the initiation of the study, all investigators from the six centres received training related to the study protocol, including the screening process, definitions of underlying diseases, and the format of the CRF. After data were collected, CRFs were reviewed by a trained researcher to ensure completeness and data quality.

### Data collection

The data were collected and included information on patient and disease characteristics, initial oxygenation strategy, laboratory and microbiological data (blood, nasopharyngeal swabs, sputum, and/or bronchoalveolar lavage samples; bacterial or fungal cultures; viral nucleic acid detection; and antibiotic susceptibility patterns), associated organ dysfunction, and patient outcomes at hospital discharge.

### Microbiological methods

Microbiological samplings were performed, bronchoalveolar lavage (BAL) or sputum samples were obtained by the treating physicians, and microorganisms were identified and tested for drug susceptibilities. Bronchoscopic examinations were performed according to general guidelines. Lidocaine spray was applied to the upper airway and carina as a local anaesthetic, and airways were thoroughly examined. BAL was performed by administering 60–120 mL of sterile saline solution 2–4 times into the distal bronchial tree, either at the affected lobe or in the middle lung lobe with more radiographic abnormalities. BAL specimens were aliquoted and immediately transported to laboratories. Sputum, BAL samples, or nasopharyngeal swabs were used for atypical pathogen and viral polymerase chain reaction (PCR) amplification tests. Reverse-transcription real time PCR (RT-PCR) (Shanghai Zhijiang Biological Technology, China) was used to detect respiratory viruses including CMV, RSV, IFV types A and B, PIV, HRV, human metapneumovirus (HMPV), and adenovirus (AdV) and *Mycoplasma pneumoniae*, *Chlamydia pneumoniae*, *Legionella pneumophila*, and *Pneumocystis jirovecii* (PCP) in nasopharyngeal swab, sputum, endotracheal aspirate (ETA), or BAL fluid sample. In addition, sputum, ETA, and BAL samples were cultured to identify the presence of bacterial and fungal organisms; the Platelia *Aspergillus* test was used for galactomannan detection (Bio-Rad Laboratories, Marnes-la-Coquette, France).

### Pathogen-specific diagnostic criteria

To diagnose pneumonia caused by *Aspergillus*, one or more of the following criteria were required: (1) histopathologic or direct microscopic evidence of dichotomous septate hyphae with a positive culture for *Aspergillus* from tissue sample, (2) a positive *Aspergillus* culture from BAL fluid sample, (3) a galactomannan optical index in BAL fluid ≥1, (4) a galactomannan optical index in serum ≥0.5; (5) *Aspergillus* species identified on culture and microscopically [13, 14].

The diagnosis of *Pneumocystis jirovecii* pneumonia (PCP) required one of the following: (1) high-resolution computed tomography imaging showing diffuse ground glass opacity with patchy distribution; (2) microscopic examination of the respiratory sample revealing the presence of *Pneumocystis* cystic or trophic forms; or (3) a positive PCR test result for *Pneumocystis* [15].

Coinfection was considered if bacteria or fungi were isolated from lower respiratory tract specimens (qualified sputum, endotracheal aspirate, and BAL) within 48 h of hospitalisation. A nosocomial infection was diagnosed when patients showed clinical signs or symptoms of pneumonia or bacteraemia and had a positive culture of a new pathogen obtained from lower respiratory tract specimens and/or blood samples taken ≥48 h after admission.

### Statistical analysis

The demographics, clinical characteristics, and pathogen testing results are expressed as the mean (± standard deviation), median (interquartile range), or number (percentage). Group comparisons were conducted using Student’s t-test or the Wilcoxon rank-sum test for continuous variables with and without normal distributions, respectively. Categorical variables of the two groups were compared using the χ^2^ test.

Statistical analyses were performed using SPSS version 19.0 (SPSS, Inc., Chicago, Illinois). All tests were two-sided, and *P*-values < 0.05 were considered statistically significant.

### Patient and public involvement

No patient or the public were involved in the development of the research question, study design, recruitment, and the conduct of the study.

## Results

A total of 860 adult patients with positive respiratory viral nucleic acid test results were selected. After excluding patients with upper respiratory tract infections (*n* = 24) and those who failed to meet the diagnostic criteria for pneumonia (*n* = 30), 806 patients with viral pneumonia were included in the final analysis. These included 370 immunocompromised and 436 immunocompetent patients. Approximately 34.3% (127/370) of the immunocompromised patients were women with a median age of 60 years. The main presenting symptoms were fever (74.6%), cough (92.4%), and dyspnoea (66.2%). The most common underlying immune-related diseases were connective tissue disease (36.2%), interstitial lung disease (44.6%), solid-organ transplantation (16.2%), and nephrotic syndrome or chronic glomerulonephritis (12.4%). D-dimer levels, pneumonia severity index (PSI) scores, rates of non-invasive mechanical ventilation, septic shock, and in-hospital mortality were higher in the immunocompromised group than in the immunocompetent group (*P* < 0.05) (Table 1).

**Table 1:** Clinical characteristics of viral pneumonia between immunocompetent and immunocompromised group

Variables	Total, *N* = 806	Immunocompromised group, *n* = 370	Immunocompetent group, *n* = 436	*P*-Value
Sex, female, n (%)	290 (36.0)	127 (34.3)	163 (37.4)	0.367
Age, median (IQR)	62.0 (49.0–71.0)	60.0 (49.0–68.0)	63.0 (49.3–75.0)	0.003
Symptoms and signs, n (%)				
Fever	608 (75.4)	276 (74.6)	332 (76.1)	0.610
Cough	764 (94.8)	342 (92.4)	422 (96.8)	0.006
Expectoration	732 (90.8)	322 (87.0)	410 (94.0)	0.001
Dyspnea	542 (67.2)	245 (66.2)	297 (68.1)	0.566
Laboratory examination				
White blood cell, × 10^9^/L (IQR)	7.85 (5.62–11.34)	8.20 (5.73–11.71)	7.55 (5.43–10.91)	0.086
Neutrophils, ×10^9^/L (IQR)	6.17 (3.82–9.22)	6.73 (4.31–9.80)	5.52 (3.51–8.95)	0.014
Lymphocyte, ×10^9^/L (IQR)	0.95 (0.56–1.52)	0.84 (0.45–1.40)	1.03 (0.61–1.58)	0.001
Persistent lymphocytopenia	319 (39.6)	177 (47.8)	142 (32.6)	< 0.001
Mean hemoglobin±SD, g/L	117.8 ± 24.5	110.6 ± 23.6	123.9 ± 23.6	< 0.001
Mean albumin±SD, g/L	34.4 ± 6.6	33.5 ± 6.6	35.2 ± 6.5	< 0.001
Lactate dehydrogenase, U/L	302 (217–501)	357 (245–555)	263 (199–454)	< 0.001
Blood urea nitrogen, mmol/L	5.95 (4.18–9.61)	6.69 (4.61–11.62)	5.39 (3.90–7.89)	< 0.001
D-Dimer, mmol/L	1.61 (0.69–4.32)	2.06 (0.84–9.42)	1.37 (0.58–3.10)	< 0.001
Procalcitonin, ng/ml	0.31 (0.17–0.82)	0.32 (0.16–0.72)	0.31 (0.18–0.94)	0.372
Oxygenation index	203 (118–289)	186 (113–289)	209 (126–292)	0.401
Severe pneumonia index score	78 (59–103)	83 (62–107)	75 (56–99)	0.001
CURB65 score > 1	261 (32.4)	117 (31.6)	144 (33.0)	0.671
Underlying Diseases, n (%)				
Without underlying disease	106 (13.2)	0 (0)	106 (24.3)	< 0.001
Diabetes mellitus	194 (24.1)	103 (27.8)	91 (20.9)	0.021
Tumor	62 (7.7)	41 (11.1)	21 (4.8)	0.001
Connective tissue disease^a^	140 (17.4)	134 (36.2)	6 (1.4)	< 0.001
Interstitial lung disease	210 (26.1)	165 (44.6)	45 (10.3)	< 0.001
Bronchiectasis	28 (3.5)	6 (1.6)	22 (5.0)	0.008
Bronchial asthma	17 (2.1)	6 (1.6)	11 (2.5)	0.375
Chronic obstructive pulmonary disease	85 (10.5)	24 (6.5)	61 (14.0)	0.001
Cirrhosis	5 (0.6)	5 (1.4)	0 (0)	0.015
Leukemia	7 (0.9)	7 (1.9)	0 (0)	0.004
Lymphoma	17 (2.1)	16 (4.3)	1 (0.2)	< 0.001
Nephrotic syndrome or chronic glomerulonephritis	50 (6.2)	46 (12.4)	4 (0.9)	< 0.001
Chronic renal failure	45 (5.6)	29 (7.8)	16 (3.7)	0.003
After bone marrow or hematopoietic stem cell transplantation	5 (0.6)	5 (1.4)	0 (0)	0.015
Solid organ transplant	60 (7.4)	60 (16.2)	0 (0)	< 0.001
Current smoker or ex-smoker	287 (35.6)	128 (34.6)	159 (36.5)	0.599
Bronchoalveolar lavage, n (%)	609 (75.6)	271 (73.2)	338 (77.5)	0.159
Treatment, before admission, n (%)				
Antibiotics	665 (82.5)	280 (75.7)	385 (88.3)	< 0.001
Antiviral drugs	164 (20.3)	83 (22.4)	81 (18.6)	0.176
Treatment, during hospitalization, n (%)				
Anti - *Pseudomonas aeruginosa* drugs	627 (77.8)	295 (79.7)	332 (76.1)	0.223
Voriconazole or caspofungin	288 (35.7)	181 (48.9)	107 (24.5)	< 0.001
Ganciclovir	254 (31.5)	221 (59.7)	33 (7.6)	< 0.001
Trimethoprim	207 (25.7)	193 (52.2)	14 (3.2)	< 0.001
Complications, n (%)				
Noninvasive ventilation	146 (18.1)	90 (24.3)	56 (12.8)	< 0.001
Invasive mechanical ventilation	234 (29.0)	98 (26.5)	136 (31.2)	0.183
Mechanical ventilation	310 (38.5)	141 (38.1)	169 (38.8)	0.982
Respiratory failure during admission	397 (49.3)	186 (50.3)	211 (48.4)	0.379
ICU admission	349 (43.3)	156 (42.2)	193 (44.3)	0.532
Septic shock during hospitalization	170 (21.1)	91 (24.6)	79 (18.1)	0.025
Extracorporeal membrane oxygenation	58 (7.2)	24 (6.5)	34 (7.8)	0.922
Hospital mortality	180 (22.3)	98 (26.5)	82 (18.8)	0.008

^a^Connective tissue disorders: rheumatoid arthritis, systemic lupus erythematosus, dermatomyositis, polymyositis, systemic sclerosis, Sjogren’s syndrome, etc.

**Fig. 1:**
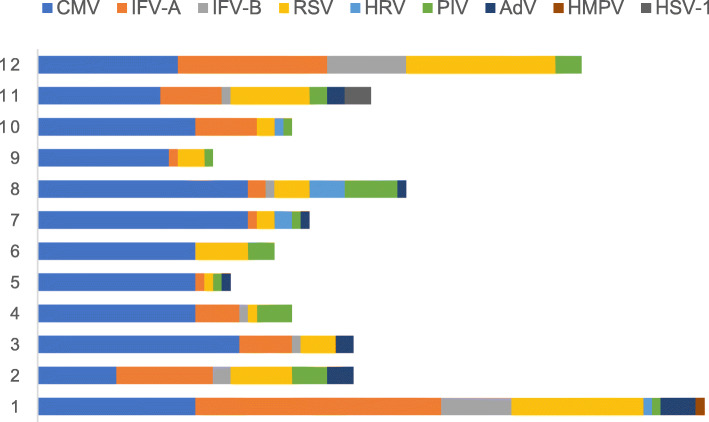
Virus detection of immunocompromised hosts in different months

**Fig. 2:**
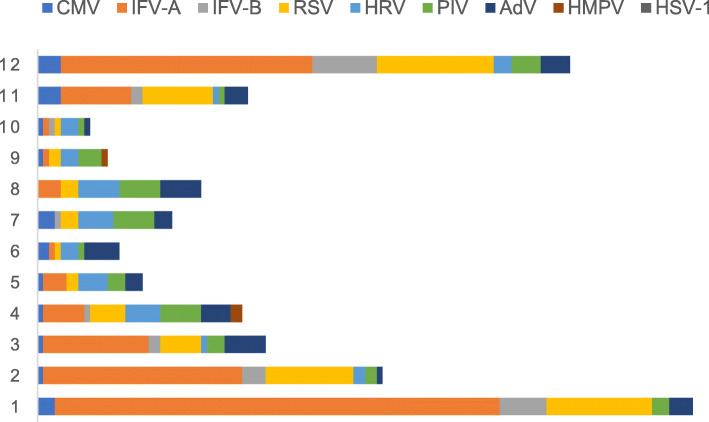
Virus detection of immunocompetent hosts in different months

During the influenza season (November, December, January, and February), an increase in the number of IFV infection cases (22.4%) was found in the immunocompromised group, followed by CMV (15.4%) and RSV (13.0%) infection cases. In the immunocompetent group, IFV (43.5%) was most frequently detected, followed by RSV (14.9%). During the non-influenza season, CMV (42.7%) was the main virus detected in the immunocompromised group. However, in the immunocompetent group, there was no dominant virus; the order of detection was as follows: IFV (9.4%), PIV (7.6%), AdV (7.3%) HRV (7.1%), and RSV (5.7%) (Table 2 and Figs. 1 and 2). Regarding coinfections in immunocompromised patients, PCP (22.4%), *Aspergillus* (14.1%) and bacteria (13.8%) were most frequent, with *Klebsiella pneumoniae* (4.1%), *Pseudomonas aeruginosa* (3.0%), and *Staphylococcus aureus* (3.0%) being the most common bacteria. In the immunocompetent group, *Aspergillus* (10.8%), bacteria (7.1%), and *Mycoplasma* (5.3%) were the dominant pathogens, with *S. aureus* (2.5%), *K. pneumoniae* (2.1%), and *Streptococcus pneumoniae* (1.1%) being the dominant bacteria. Among the secondary nosocomial bacterial infections, *Acinetobacter baumannii*, *P. aeruginosa,* and *K. pneumoniae* were most commonly detected as causative agents (Table 2). The CMV infection group had more patients with nephrotic syndrome and high rates of PCP infection and ground glass shadows on computed tomography (CT) (*P* < 0.05). In the non-IFV group, there were fewer patients who required non-invasive ventilator use and intensive care unit treatment than other groups. Further, the non-IFV group was associated with a lower in-hospital mortality rate than CMV and two-or-more viruses’ groups. However, patients with IFV and non-IFV infection in immunocompromised patients had similar disease severity and prognosis (Table 3).

**Table 2:** The pathogen results of pneumonia between immunocompetent and immunocompromised group

Variables, n (%)	Immunocompromised group, *n* = 370	Immunocompetent group, *n* = 436	*P*-Value
One virus	305 (82.4)	396 (90.8)	< 0.001
Two or more viruses	65 (17.6)	40 (9.2)	< 0.001
Influenza season			
Cytomegalovirus	57 (15.4)	12 (2.8)	< 0.001
Influenza A virus	63 (17.0)	165 (37.8)	< 0.001
Influenza B virus	20 (5.4)	25 (5.7)	0.840
Rhinovirus	1 (0.3)	6 (1.4)	0.092
Respiratory syncytial virus	48 (13.0)	65 (14.9)	0.430
Adenovirus	9 (2.4)	14 (3.2)	0.508
Parainfluenza virus	10 (2.7)	11 (2.5)	0.873
Human metapneumovirus	1 (0.3)	0 (0)	0.277
HSV-1	3 (0.8)	0 (0)	0.060
Non-influenza season			
Cytomegalovirus	158 (42.7)	10 (2.3)	< 0.001
Influenza A virus	23 (6.2)	36 (8.3)	0.268
Influenza B virus	3 (0.8)	5 (1.1)	0.632
Rhinovirus	7 (1.9)	31 (7.1)	< 0.001
Respiratory syncytial virus	21 (5.7)	25 (5.7)	0.972
Adenovirus	5 (1.4)	32 (7.3)	< 0.001
Parainfluenza virus	17 (4.6)	33 (7.6)	0.081
Human metapneumovirus	0 (0)	3 (0.7)	0.110
Pathogenic types of coinfections	204 (55.1)	101 (23.2)	< 0.001
Bacteria	51 (13.8)	31 (7.1)	0.002
*Streptococcus pneumoniae*	1 (0.3)	5 (1.1)	0.149
*Streptococcus constellatus*	1 (0.3)	0 (0)	0.277
*Haemophilus influenzae*	1 (0.3)	0 (0)	0.277
*Staphylococcus aureus*	11 (3.0)	11 (2.5)	0.696
*Escherichia coli*	3 (0.8)	1 (0.2)	0.242
*Enterobacter aerogenes*	0 (0)	1 (0.2)	0.357
*Enterobacter cloacae*	2 (0.5)	0 (0)	0.470
*Klebsiella pneumoniae*	15 (4.1)	9 (2.1)	0.098
*Pseudomonas*	11 (3.0)	4 (0.9)	0.031
*Proteus mirabilis*	2 (0.5)	0 (0)	0.470
*Acinetobacter*	2 (0.5)	0 (0)	0.470
*Nocardia*	2 (0.5)	0 (0)	0.470
*Atypical*	11 (3.0)	23 (5.3)	0.105
*Mycoplasma pneumoniae*	6 (1.6)	23 (5.3)	0.006
*Legionella*	5 (1.4)	0 (0)	0.015
Pneumocystis	83 (22.4)	0 (0)	< 0.001
Aspergillus	52 (14.1)	47 (10.8)	0.158
*Mycobacterium tuberculosis*	6 (1.6)	0 (0)	0.008
Non-tuberculosis mycobacteria	1 (0.3)	0 (0)	0.277
Pathogens in nosocomial infection	134 (36.2)	168 (38.5)	0.498
*Acinetobacter*	31 (8.4)	52 (11.9)	0.099
*Pseudomonas*	32 (8.6)	41 (9.4)	0.710
*Klebsiella pneumoniae*	14 (3.8)	17 (3.9)	0.932
*Burkholderia*	11 (3.0)	17 (3.9)	0.474
*Enterococcus*	6 (1.6)	2 (0.5)	0.097
*Enterobacter cloacae*	3 (0.8)	0 (0)	0.060
*Escherichia coli*	4 (1.1)	1 (0.2)	0.125
*Proteus mirabilis*	0 (0)	2 (0.5)	0.192
*Stenotrophomonas maltophilia*	4 (1.1)	11 (2.5)	0.131
*Corynebacterium striatum*	6 (1.6)	11 (2.5)	0.375
*Staphylococcus aureus*	4 (1.1)	0 (0)	0.030
*Rolstonia mannitolytica*	1 (0.3)	5 (1.1)	0.149
*Other bacteria*	5 (1.4)	3 (0.7)	0.344
Aspergillus	12 (3.2)	6 (1.4)	0.074
Trichosporon asahii	1 (0.3)	0 (0)	0.277
Only one virus	123 (33.2)	259 (59.4)	< 0.001
>one organism	247 (66.8)	177 (40.6)	< 0.001

*HSV-1* herpes simplex virus type 1

**Table 3:** Comparative analysis of different viral pneumonia in immunocompromised patients

Variables	CMV *N* = 162	IFV *N* = 65	Non-IFV *N* = 79	≥two viruses *N* = 64	*P*-Value
Female, n (%)	59 (36.4)	18 (27.7)	30 (38.0)	20 (31.3)	0.509
Age, median (IQR), years	60.0 (47.0, 68.3)	63.0 (54.0, 69.0)	59.0 (47.0, 68.0)	60.0 (50.3, 67.0)	0.616
Symptoms and signs, n (%)					
Fever	138 (85.2)	48 (73.9)	48 (60.8)	42 (65.6)	< 0.001
Cough	138 (85.2)	63 (96.9)	79 (100.0)	62 (96.9)	< 0.001
Expectoration	122 (75.3)	63 (96.9)	77 (97.5)	60 (93.8)	< 0.001
Dyspnea	108 (66.7)	43 (66.2)	51 (64.6)	43 (67.2)	0.987
Underlying Diseases, n (%)					
Connective tissue disease	69 (42.6)	19 (29.2)	21 (26.6)	25 (39.1)	0.054
Interstitial lung disease	61 (37.7)	31 (47.7)	43 (54.4)	30 (46.9)	0.084
Diabetes mellitus	43 (26.5)	18 (27.7)	22 (27.9)	20 (31.3)	0.917
Tumor	19 (11.7)	7 (10.8)	11 (13.9)	4 (6.3)	0.524
Bronchial asthma	6 (3.7)	0 (0)	0 (0)	0 (0)	0.050
COPD	13 (8.0)	6 (9.2)	3 (3.8)	2 (3.1)	0.311
Leukemia	2 (1.2)	0 (0)	4 (5.1)	1 (1.6)	0.114
Lymphoma	5 (3.1)	2 (3.1)	5 (6.3)	4 (6.3)	0.535
After bone marrow or HSCT	2 (1.2)	0 (0)	1 (1.3)	2 (3.1)	0.490
Nephrotic syndrome or chronic glomerulonephritis	36 (22.2)	2 (3.1)	4 (5.1)	4 (6.3)	< 0.001
Solid organ transplant	7 (4.3)	17 (26.2)	23 (29.1)	13 (20.3)	< 0.001
Cirrhosis	0 (0)	3 (4.6)	1 (1.3)	1 (1.6)	0.059
Laboratory examination					
White blood cell, ×10^9^/L (IQR)	8.50 (5.70, 12.52)	7.95 (5.08, 11.07)	7.57 (5.69, 11.47)	8.50 (6.35, 11.45)	0.587
Neutrophils, ×10^9^/L (IQR)	7.08 (4.52, 10.94)	6.80 (3.80, 9.24)	5.69 (3.51, 8.81)	6.90 (4.86, 9.77)	0.081
Lymphocyte, × 10^9^/L (IQR)	0.73 (0.41, 1.40)	0.81 (0.41, 1.31)	1.11 (0.60, 1.83)	0.80 (0.45, 1.32)	0.048
Persistent lymphocytopenia	84 (51.9)	32 (49.2)	28 (35.4)	33 (51.6)	0.097
D-Dimer, mg/L	1.78 (0.78, 3.08)	1.52 (0.58, 3.09)	1.12 (0.55, 2.68)	1.34 (0.60, 2.57)	0.288
Lactate dehydrogenase, U/L	395.5 (255.8, 590.0)	325.0 (228.0, 482.0)	300.0 (206.0, 430.0)	386.0 (276.0, 553.9)	0.007
Oxygenation index	184.2 (113.5, 286.0)	285.7 (154.1375.9)	244.1 (96.3, 277.1)	122.4 (92.5, 272.8)	0.067
Severe pneumonia index score	75.0 (58.0, 107.0)	79.0 (60.0, 99.0)	79.0 (61.0, 104.0)	80.5 (57.8, 105.3)	0.508
CURB65 score > 1	55 (34.0)	25 (38.5)	19 (24.1)	18 (28.1)	0.234
Imaging features, n (%),24 missing					
Consolidation or mass	71 (43.8)	24 (36.9)	39 (49.4)	34 (53.1)	0.176
Ground-glass opacity	99 (61.1)	30 (46.2)	42 (53.2)	35 (54.7)	0.004
Viral-PCP co-infection	64 (39.5)	4 (6.2)	3 (3.8)	7 (10.9)	< 0.001
Viral-aspergillus co-infection	16 (9.9)	9 (13.8)	12 (15.2)	15 (23.4)	0.069
Viral-bacteria co-infection	22 (13.6)	7 (10.8)	10 (12.7)	9 (14.1)	0.939
Viral-atypical co-infection	6 (3.7)	1 (1.5)	3 (3.8)	1 (1.6)	0.708
Nosocomial bacterial infection	36 (22.2)	17 (26.2)	17 (21.5)	22 (34.4)	0.237
Complications, n (%)					
Noninvasive ventilation	54 (33.3)	10 (15.4)	9 (11.4)	17 (26.6)	0.001
Invasive mechanical ventilation	45 (27.8)	20 (30.8)	16 (20.3)	19 (29.7)	0.462
Respiratory failure	91 (56.2)	33 (50.8)	26 (32.9)	36 (56.3)	0.001
ICU care	89 (54.9)	22 (33.8)	19 (24.1)	26 (40.6)	< 0.001
Septic shock	40 (24.7)	17 (26.2)	14 (17.7)	20 (31.3)	0.305
Extracorporeal membrane oxygenation	4 (2.5)	7 (10.8)	7 (8.9)	6 (9.4)	0.268
In-hospital mortality	50 (30.9)	14 (21.5)	12 (15.2)	22 (34.4)	0.022^a^

*IFV* influenza A virus, influenza B virus; *Non-IFV virus* respiratory syncytial virus (RSV), *HPIV* human parainfluenza virus, *HRV* human rhinovirus, *ADV* adenovirus and *HSV-1* herpes simplex virus type 1, *HSCT* hematopoietic stem cell transplantation, *COPD* Chronic obstructive pulmonary disease.

^a^The in-hospital mortality between non-IFV and IFV patients was not statistically different (*P* = 0.324), but the non-IFV group was associated with a lower in -hospital rate than that of CMV group and two or more viruses' group (*P*<0.05)

Patients with nephrotic syndrome and chronic glomerulonephritis had the highest rate of CMV infection (89.1%), organ transplant patients had the highest rate of RSV infection (35.0%), patients with haematopoiesis diseases had the highest rates of AdV (22.7%) and HRV (18.2%) infections, and malignant solid patients with radiotherapy and chemotherapy had the highest rate of PIV infection (23.5%). Patients with nephrotic syndrome and chronic glomerulonephritis had a low oxygenation index and lymphocyte count, high rate of CMV and PCP infection, were more likely to require additional non-invasive ventilator use and intensive care unit treatment, and had a high in-hospital mortality rate. The in-hospital mortality rate of patients with connective tissue disease was the second highest (30%), while that of solid-organ transplantation patients was the lowest (10%) (Table 4). Viral shedding was significantly longer in immunocompromised hosts than in immunocompetent hosts (Table 5).

**Table 4:** Clinical characteristics of pneumonia with immunocompromised patients in different underlying disease

Variables	Connective tissue disease, *N* = 134	Solid organ transplant, *N* = 60	Nephrotic syndrome or chronic glomerulonephritis, *N* = 46	Hematopoiesis diseases^a^ *N* = 22	Idiopathic interstitial pneumonia, *N* = 51	Radiotherapy and chemotherapy of malignant solid tumor, *N* = 17	*P* value
Sex, female, n (%)	64 (47.8)	11 (18.3)	11 (23.9)	7 (31.8)	16 (31.4)	3 (17.6)	< 0.001
Age, median (IGR)	62.0 (45.0, 70.3)	58.0 (47.0, 63.0)	58.0 (47.8, 65.3)	55.0 (32.8, 69.5)	59.0 (53.0, 69.0)	64.0 (57.0, 67.0)	0.043
Laboratory examination							
White blood cell, × 10^9^/L (IQR)	8.59 (6.30, 11.72)	6.79 (4.47, 9.81)	8.83 (6.44, 11.97)	5.58 (3.21, 9.85)	7.85 (5.73, 11.48)	8.01 (4.21, 10.77)	0.005
Neutrophils, × 10^9^/L (IQR)	6.99 (5.05, 9.80)	4.63 (3.11, 7.70)	8.20 (5.2, 10.9)	4.19 (1.89, 7.51)	6.45 (4.60, 9.58)	6.73 (2.91, 8.30)	0.001
Lymphocyte, × 10^9^/L (IQR)	0.81 (0.44, 1.45)	0.95 (0.36, 1.62)	0.62 (0.33, 0.96)	0.70 (0.22, 1.34)	1.09 (0.70, 1.83)	0.80 (0.46, 1.21)	0.039
Oxygenation index	212.4 (116.8, 291.8)	244.1 (142.4, 338.1)	122.0 (78.6, 206.2)	225.8 (116.1, 368.2)	209.2 (111.3, 328.5)	327.4 (296.2, 413.6)	0.026
Severe pneumonia index score	76.0 (50.8, 103.0)	83.0 (64.3, 100.0)	89.5 (66.8, 119.0)	87.5 (59.3, 119.0)	79.0 (63.0, 91.0)	107.0 (80.0, 125.0)	0.018
CURB65 score > 1	37 (27.6)	17 (28.3)	19 (41.3)	4 (18.2)	15 (29.4)	6 (35.3)	0.421
Imaging features, n (%)	126 (94.0)	59 (98.3)	37 (80.4)	18 (81.8)	51 (100.0)	15 (88.2)	–
Consolidation or mass	86 (64.2)	26 (43.3)	27 (58.7)	5 (22.7)	38 (74.5)	8 (47.1)	< 0.001
Ground-glass opacity	65 (48.5)	22 (36.7)	21 (45.7)	11 (50.0)	18 (35.3)	10 (58.8)	0.049
CMV	89 (66.4)	18 (30.0)	41 (89.1)	11 (50.0)	25 (49.0)	7 (41.2)	< 0.001
IFV-A	24 (17.9)	18 (30.0)	5 (10.9)	3 (13.6)	12 (23.5)	6 (35.3)	0.086
IFV-B	9 (6.7)	5 (8.3)	0 (0)	0 (0)	5 (9.8)	2 (11.8)	0.229
RSV	26 (19.4)	21 (35.0)	3 (6.5)	2 (9.1)	14 (27.5)	0 (0)	0.001
AdV	3 (2.2)	3 (5.0)	0 (0)	5 (22.7)	0 (0)	1 (5.9)	< 0.001
HRV	2 (1.5)	1 (1.7)	0 (0)	4 (18.2)	0 (0)	0 (0)	< 0.001
PIV	9 (6.7)	6 (10.0)	0 (0)	3 (13.6)	3 (5.9)	4 (23.5)	0.035
Viral-PCP co-infection	30 (22.4)	3 (5.0)	24 (52.2)	3 (13.6)	9 (17.6)	4 (23.5)	< 0.001
Viral-aspergillus co-infection	13 (9.7)	17 (28.3)	5 (10.9)	1 (4.5)	6 (11.8)	2 (11.8)	0.010
Viral-bacteria co-infection	16 (11.9)	12 (20.0)	6 (13.0)	1 (4.5)	2 (3.9)	3 (17.6)	0.134
Viral-atypical co-infection	4 (3.0)	2 (3.3)	3 (6.5)	1 (4.5)	0 (0)	0 (0)	0.518
Nosocomial bacterial infection	26 (19.4)	25 (41.7)	12 (26.1)	5 (22.7)	11 (21.6)	2 (11.8)	0.021
Complications, n (%)							
NIV	41 (30.6)	8 (13.3)	17 (37.0)	4 (18.2)	15 (29.4)	2 (11.8)	0.035
IMV	41 (30.6)	9 (15.0)	12 (26.1)	3 (13.6)	17 (33.3)	1 (5.9)	0.033
Respiratory failure	78 (58.2)	21 (35.0)	24 (52.2)	6 (27.3)	28 (54.9)	6 (35.3)	0.004
ICU care	66 (49.3)	10 (16.7)	28 (60.9)	8 (36.4)	25 (49.0)	2 (11.8)	< 0.001
Septic shock	31 (23.1)	12 (20.0)	17 (37.0)	3 (13.6)	11 (21.6)	5 (29.4)	0.256
ECMO	8 (6.0)	4 (6.7)	3 (6.5)	1 (4.5)	7 (13.7)	0 (0)	0.297
In-hospital mortality	40 (30.0)	6 (10.0)	18 (39.1)	3 (13.6)	13 (25.5)	4 (23.5)	0.011

*NIV* Noninvasive ventilation, *IMV* Invasive mechanical ventilation, *ECMO* Extracorporeal membrane oxygenation

^a^Hematopoiesis diseases: Leukemia, lymphoma, bone marrow or hematopoietic stem cell transplantation

**Table 5:** viral shedding in of different groups

Variables	Viral shedding in immunocompromised group(d)	Viral shedding in immunocompetent group(d)	*P* value
IFV	12.0 (6.5, 26.5)	8.5 (5.0, 13.0)	0.022
RSV	14.0 (6.0, 30.0)	6.5 (3.0, 14.0)	0.024

## Discussion

This study was a large-scale, multicentre, retrospective study of the aetiology of and clinical risk factors for CAP in immunocompromised patients. The main findings were as follows: (1) The disease severity and in-hospital mortality rate of immunocompromised patients were higher than those of immunocompetent patients; (2) during the influenza and non-influenza seasons, the distribution of viruses in the immunocompromised group differed; (3) among the coinfections of immunocompromised patients, PCP was the main pathogen, followed by *Aspergillus* and bacteria, and in the immunocompetent group, *Aspergillus* was the most common pathogen, followed by bacteria and *Mycoplasma*; (4) the in-hospital mortality rate of the non-IFV infection group was lower than those of the CMV group and the two-or-more viruses group, but had similar prognosis with IFV group; (5) the type of virus infection varied according to the underlying diseases detected; (6) viral shedding was significantly longer in immunocompromised hosts than in immunocompetent hosts.

In recent years, several studies have focused on respiratory virus infection in patients after haematopoietic cell transplantation (HCT) [16–21]. Sachiko studied HRV in the lower respiratory tract of patients with HCT and found that 55% of patients had coinfections and that the 90-day mortality rate was 41% [16], which was similar to that of lower respiratory tract infections caused by RSV, PIV, or IFV [17–19]. Among the immunocompromised patients with IFV pneumonia, approximately 60% had an associated infection with at least one other organism, and the mortality rate among these patients was 15–30% [20]. The mortality rate among haematologic malignancy patients with RSV is approximately 18%, and in HCT recipients who developed RSV lower respiratory tract infections, it can be as high as 83% [21]. Similarly, our study showed that the disease severity and in-hospital mortality (26.5% vs 18.8%) of immunocompromised patients were higher than those of immunocompetent patients.

CMV, especially with PCP coinfection, has a high mortality rate in immunocompromised patients [22, 23]. However, at present, there are few comparative studies examining CMV and other respiratory viruses. Our findings indicated that during the influenza season, IFV, CMV, and RSV were the main viruses detected in immunocompromised hosts, while during the non-influenza season, we need to pay attention to CMV, IFV, PIV, AdV, HRV and RSV as these were more readily detected. Non-CMV viral infections may also exist with a PCP coinfection, albeit less frequent. Comparably we found no difference in the rate of virus-*Aspergillus* coinfections irrespective of the type of viral infection [13, 24].

The disease severity in, complications in, and outcomes of immunocompetent patients with CAP were similar between IFV- and non-IFV-related respiratory diseases [25–27]. We found that the in-hospital mortality rate was significantly higher in immunocompromised patients with CMV or two-or-more viral infections than the non-IFV infections. This suggests that when a viral infection is suspected in an immunocompromised patient, healthcare providers should also determine the presence of CMV and other viral aetiologies, as early diagnosis and treatment are essential in improving the outcomes. In addition, the highest mortality rate was observed among patients with nephrotic syndrome or chronic glomerulonephritis, for which there was a higher rate of CMV and PCP infection. This indicates that routinely screening for PCP and CMV infections should be considered for this group of patients. Moreover, the higher incidence of CMV and PCP and mortality rates associated with nephrotic syndrome patients may be related to the lack of routine prevention of infection when using immunosuppressants or glucocorticoids.

It has been suggested that viral respiratory infections in immunocompromised patients involve persistent viral shedding, rendering these patients contagious for prolonged periods [28–30]. Memoli et al. reported that the viral shedding period of immunocompromised patients was longer than that of immunocompetent patients with IFV pneumonia (19.04 vs. 6.38 days, respectively; *P* < 0.05) [29]. Virus detection for ≥30 days was reported in 29% of infected patients with haematological disorders [28]. In this study, we demonstrated that both influenza A virus subtype H1N1 and RSV infections had a longer viral shedding period in immunocompromised hosts, which made it necessary to extend the duration of antiviral therapy.

There were some limitations to this study. First, it had a retrospective design and might not have included all patients. Second, as it was a multicentre research, not every patient with pneumonia underwent a full array of pathogen testing. Therefore, pathogen identification and diagnosis could have been incomplete. Third, many patients had been previously administered antibiotics. Despite these limitations, our results were consistent with the literature and provide a detail insight into the clinical and pathogenic characteristics and outcomes of different viral infections in immunocompromised hosts.

## Conclusions

Immunocompromised patients have high frequencies of coinfections, nosocomial infections, and mortality rates. A longer viral shedding duration may lead to a prolonged period of infectivity.

## Supplementary Information

**Additional file 1: Supplementary Table 1**: virus detection in immunocompetent and immunocompromised group.

## Data Availability

The datasets used and/or analysed during the current study are available from the corresponding author on reasonable request.

## Abbreviations

CAP: Community-acquired pneumonia
CMV: Cytomegalovirus
Flu A: Influenza A virus
Flu B: Influenza B virus
PIV: Parainfluenza virus
RSV: Respiratory syncytial virus
AdV: Adenovirus
HRV: Rhinovirus
HMPV: Human metapneumovirus
HSV: Herpes virus
PCP: *Pneumocystis jirovecii* pneumonia
ETA: Endotracheal aspirate
BAL: Bronchoalveolar lavage
PSI: Pneumonia severity index
HCT: Haematopoietic cell transplantation
RT-PCR: Reverse-transcription real time polymerase chain reaction
