# Supplementary material for: Characteristics of viral pneumonia in non-HIV immunocompromised and immunocompetent patients: a retrospective cohort study
Source: BMC Infect Dis. 2021 Aug 6;21:767. doi: 10.1186/s12879-021-06437-5 (PMC8343364; doi:10.1186/s12879-021-06437-5)
Supplement: Supplementary file 1 — Additional file 1: Supplementary Table 1: virus detection in immunocompetent and immunocompromised group. [file 12879_2021_6437_MOESM1_ESM.docx]

**supplementary table1: virus detection in immunocompetent and immunocompromised group**

| Variables | Number of virus detection in immunocompromised patients | Positive | Number of virus detection in immunocompetent patients | Positive |
| --- | --- | --- | --- | --- |
| Cytomegalovirus | 357 | 215(60.2) | 368 | 22 (6.0) |
| Influenza A virus | 290 | 79 (27.2) | 428 | 201 (47.0) |
| Influenza B virus | 290 | 23 (7.9) | 428 | 30 (70.1) |
| Rhinovirus | 78 | 8 (10.3) | 97 | 40 (41.2) |
| Respiratory syncytial virus | 236 | 71 (30.1) | 411 | 90 (21.9) |
| Adenovirus | 184 | 14 (7.7) | 311 | 46 (14.8) |
| [Parainfluenza virus](http://www.baidu.com/link?url=rO33W2_8EggTpVzx3VNoc5oK2eAZjXKfLav1-TypKwhPcdFSL7P16Ql82MFgmD1i1N2CG_A7zaO42cntcPNphEg0FaMlpOFse57HwxG-lvrvSI_QTPFMP4VM_TIEzeh0&wd=&eqid=b48e905f0008f140000000025de1ffe9) | 169 | 27 (16.0) | 242 | 44 (18.2) |
| Human metapneumovirus | 78 | 1 (1.3) | 97 | 3 (3.1) |
| HSV-1 | 20 | 3 (15.0) | 2 | 0 (0) |
